# Use of large multiconformational databases with structure-based pharmacophore models for fast screening of commercial compound collections

**DOI:** 10.1186/1758-2946-3-S1-P27

**Published:** 2011-04-19

**Authors:** A Del Rio, AJM Barbosa, F Caporuscio

**Affiliations:** 1 Dipartimento di Scienze Farmaceutiche, Università di Modena e Reggio Emilia, Via Campi 183, 41100, Modena, Italy

## 

In the last years high-throughput pharmacophore screenings have been rediscovered as an effective and rapid tool for guiding the selection of new hit compounds with predefined biological activity. This *renaissance* has also been fostered by the current possibility to generate pharmacophore hypotheses directly from crystallographic, NMR or computational models of protein-ligand complexes [1,2]. Indeed the pharmacophore notion provides a powerful way to identify and compare structural features across a large set of molecules and the possibility to screen virtually millions of compounds. At the present time pharmacophore screenings are further stimulated by the increasing number of chemical vendors that offer their catalogs of chemical compounds also for this purpose.

Unfortunately, such an advantage is in many cases blunted by the fact that an high-throughput pharmacophore screening campaign takes time during the preparation steps of compound libraries, e.g. preparing ligand structures with full hydrogen, tautomers, stereoisomers and, most importantly, conformers. The latter point is extremely important in the context of pharmacophore screenings since the matching of a ligand molecule to a pharmacophore hypothesis is dependent upon the molecular conformation of the ligand. Furthermore, there is no specific way to predict what conformations are the biologically active ones for a given biological target. To address these issues we have recently introduced CoCoCo, a suite of free muticonformational databases that can be used for such pharmacophore screenings [3,4].

Here we will present how these ready-to-use chemical databases, that bear multiconformational information for each ligand, may provide a straightforward and time-effective way to select candidate active compounds. Different cases will be analyzed to highlight how different factors, e.g. pharmacophore hypotheses and conformational states, may influence the outcome of high-throughput screenings. Finally, a proof-of-concept experimental study that strongly supports these approaches will be presented.
